# Rare variants in dynein heavy chain genes in two individuals with *situs inversus* and developmental dyslexia: a case report

**DOI:** 10.1186/s12881-020-01020-2

**Published:** 2020-05-01

**Authors:** Andrea Bieder, Elisabet Einarsdottir, Hans Matsson, Harriet E. Nilsson, Jesper Eisfeldt, Anca Dragomir, Martin Paucar, Tobias Granberg, Tie-Qiang Li, Anna Lindstrand, Juha Kere, Isabel Tapia-Páez

**Affiliations:** 1 Department of Biosciences and Nutrition, Karolinska Institutet, Hälsovägen 7, 141 83 Huddinge, Sweden; 2 Stem Cells and Metabolism Research Program (STEMM), University of Helsinki, Helsinki, Finland; 3 Folkhälsan Institute of Genetics, Helsinki, Finland; 4 Science for Life Laboratory, Department of Gene Technology, KTH-Royal Institute of Technology, Solna, Sweden; 5 Department of Women’s and Children’s Health, Karolinska Institutet, Solna, Sweden; 6 Center for Molecular Medicine, Karolinska Institutet, Stockholm, Sweden; 7 Department of Immunology, Genetics and Pathology, Uppsala University, Uppsala, Sweden; 8 Department of Biomedical Engineering and Health Systems, School of Engineering Sciences in Chemistry, Biotechnology and Health, KTH Royal Institute of Technology, Huddinge, Sweden; 9 Department of Molecular Medicine and Surgery, Karolinska Institutet, Stockholm, Sweden; 10 Science for Life Laboratory, Karolinska Institutet Science Park, Solna, Sweden; 11 Department of Pathology, Uppsala University Hospital, Uppsala, Sweden; 12 Department of Immunology, Genetics and Pathology, Uppsala University, Uppsala, Sweden; 13 Department of Clinical Neuroscience, Karolinska Institutet, Stockholm, Sweden; 14 Department of Radiology, Karolinska University Hospital, Stockholm, Sweden; 15 Department of Clinical Science, Intervention and Technology, Karolinska Institutet, Stockholm, Sweden; 16 Department of Clinical Genetics, Karolinska University Hospital, Stockholm, Sweden; 17 School of Basic and Medical Biosciences, King’s College London, Guy’s Hospital, London, UK; 18 Department of Medicine, Solna, Karolinska Institutet, Solnavägen 30, 171 76 Solna, Stockholm, Sweden

**Keywords:** Developmental dyslexia, Situs inversus, Primary ciliary dyskinesia, L-R asymmetry defects, Whole genome sequencing, SNVs, Brain imaging

## Abstract

**Background:**

Developmental dyslexia (DD) is a neurodevelopmental learning disorder with high heritability. A number of candidate susceptibility genes have been identified, some of which are linked to the function of the cilium, an organelle regulating left-right asymmetry development in the embryo. Furthermore, it has been suggested that disrupted left-right asymmetry of the brain may play a role in neurodevelopmental disorders such as DD. However, it is unknown whether there is a common genetic cause to DD and laterality defects or ciliopathies.

**Case presentation:**

Here, we studied two individuals with co-occurring *situs inversus* (SI) and DD using whole genome sequencing to identify genetic variants of importance for DD and SI. Individual 1 had primary ciliary dyskinesia (PCD), a rare, autosomal recessive disorder with oto-sino-pulmonary phenotype and SI. We identified two rare nonsynonymous variants in the dynein axonemal heavy chain 5 gene (*DNAH5)*: a previously reported variant c.7502G > C; p.(R2501P), and a novel variant c.12043 T > G; p.(Y4015D). Both variants are predicted to be damaging. Ultrastructural analysis of the cilia revealed a lack of outer dynein arms and normal inner dynein arms. MRI of the brain revealed no significant abnormalities. Individual 2 had non-syndromic SI and DD. In individual 2, one rare variant (c.9110A > G;p.(H3037R)) in the dynein axonemal heavy chain 11 gene (*DNAH11),* coding for another component of the outer dynein arm, was identified.

**Conclusions:**

We identified the likely genetic cause of SI and PCD in one individual, and a possibly significant heterozygosity in the other, both involving dynein genes. Given the present evidence, it is unclear if the identified variants also predispose to DD and further studies into the association between laterality, ciliopathies and DD are needed.

## Background

Left-right asymmetry conditions are characterized by failure of organization of the internal organs along the left-right axis [1]. Laterality is established through a process involving motile and primary cilia at the embryonic node. A number of genes causing laterality disorders when disrupted have been identified in humans [1]. About 20–25% of *situs inversus* (SI) *totalis* - a complete reversal of internal organs – individuals are also affected by primary ciliary dyskinesia (PCD) [1]. PCD (OMIM #244400) is a rare autosomal recessive disorder, caused by functional impairment of the motile cilia. PCD leads to oto-sino-pulmonary disease with the phenotypic triad chronic sinusitis, bronchiectasis and SI (Kartagener syndrome) in approximately 50% of cases [2]. PCD has heterogeneous underlying genetics and to date, mutations in more than 40 genes have been identified as causative, of which the dynein axonemal heavy chain 5 gene *DNAH5* accounts for the largest proportion of cases (28%) [2–4].

Developmental dyslexia (DD) is one of the most common neurodevelopmental disorders, affecting around 5–12% of the population, and is highly heritable [5]. The underlying neurodevelopmental causes of DD are not yet fully understood. One hypothesis is that neuronal migration disturbances during development lead to misplacement of neurons in the adult brain, resulting in changes in white and grey matter [5]. Early studies have suggested a role of brain asymmetry, for example of the planum temporale [6].

Genetic studies of DD have led to the identification of a number of dyslexia susceptibility genes, reviewed in [7]. Interestingly, some of them, namely *DYX1C1 (DNAAF4)*, *DCDC2* and *KIAA0319,* have a reported role in cilia [8–14]. In addition, loss-of-function mutations in *DYX1C1* and *DCDC2* have been found in patients showing typical ciliary deficits: *DYX1C1* in patients with PCD [15] and *DCDC2* in patients with nephronophthisis-related ciliopathy, inherited deafness and neonatal sclerosing cholangitis [16–19]. Other dyslexia candidate genes, such as *CEP63* and *PCNT* are involved in centrosome and basal body biology [20, 21].

While DD has been associated with various anatomical and functional changes in the brain [22], a number of reports have investigated brain anatomy and functionality in individuals with *situs inversus* (for example [23–27]). Interestingly, a range of neurodevelopmental disorders, such as autism, schizophrenia and DD, has been associated with laterality defects in the brain [28]. The recent discoveries about ciliary genes and DD give new perspectives on the brain asymmetry theory and neuronal ciliary signaling theory in DD [14]. It is currently unknown whether ciliary phenotypes and DD share a common genetic cause.

Here, we sought to address a potential common genetic cause underlying SI and DD. We studied two individuals with SI and/or PCD and DD, using whole genome sequencing (WGS) to determine a possible genetic cause for their phenotype. In addition, we performed brain imaging on one of the individuals to determine potential alterations in the brain associated with SI or DD.

## Case presentation

### Case 1

**Fig. 1:**
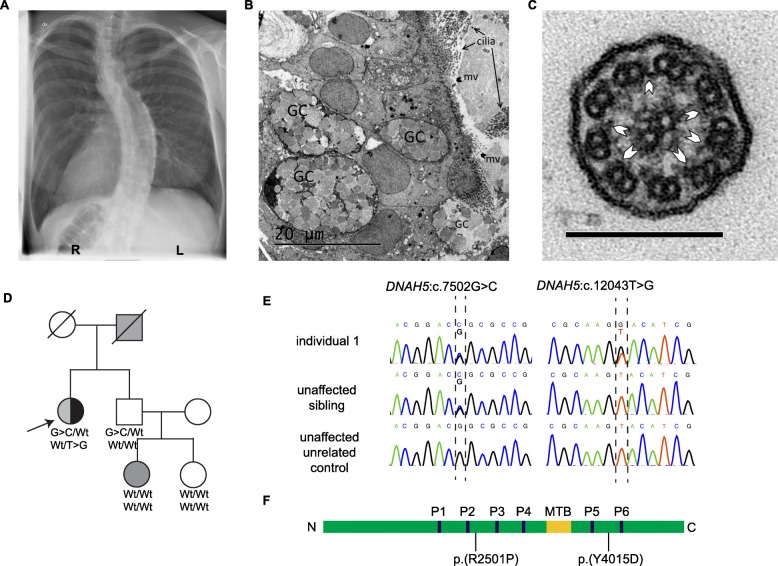
Phenotype and genetics individual 1. **a** X-Ray image showing *situs inversus* and left convex scoliosis. **b** Low magnification electron micrograph of biopsy from nasal respiratory mucosa showing hyperplasia of goblet cells (GC), reduced number of cilia and increased number of microvilli (mv). **c** High magnification electron micrograph of a cilium from the epithelial cells showing lack of outer dynein arms and normal inner dynein arms (arrowheads), normal radial spokes and central pair. Scale bar = 200 nm. **d** Pedigree. Individual 1 (arrow) has PCD (black) and DD (gray). The father and the niece are affected by DD (gray). Unaffected individuals are shown in white. The genotypes of the two variants in *DNAH5* are indicated in the affected individual and in the unaffected brother (G > C denotes c.7502G > C, T > G denotes c.12043 T > G, Wt denotes wildtype). **e** Sanger DNA sequencing chromatogram of individual 1 and controls. **f** Schematic representation of the domains of DNAH5 and localization of the amino acid substitutions p.(R2501P) and p.(Y4015D). *N*=N-terminus, C=C-terminus, MTB = microtubule-binding domain, P1-P6 = P-loops 1–6

Individual 1 is a Swedish woman affected by PCD and DD, born to non-consanguineous parents. She presented with the following symptoms of PCD: bronchiectasis, *situs inversus* (Fig. 1 a) and recurring upper and lower airway infections since birth. The findings of transmission electron microscopy of a nasal epithelial brush biopsy (see Additional file 1, Methods) included goblet cell hyperplasia, a reduced number of ciliated cells, an increased number of microvilli and sporadically distributed lymphocytes (Fig. 1 b). Characteristic for PCD was the lack of outer dynein arms (ODA) (mean 2.4 +/− 0.2 per cilium; normal interval: 7–9 ODA/cilium). The number of inner dynein arms (IDA) was within normal limits (mean 4.05 +/− 0.2; normal interval: 2–7) (Fig. 1 c). Additional diagnoses include left convex scoliosis (Fig. 1 a), attention deficit hyperactivity disorder (ADHD) and Asperger’s syndrome. Notably, the individual is left-handed (Edinburgh inventory laterality index − 60.00), and has the ability for mirror writing, a phenomenon overrepresented in dyslexics and left-handed persons [29]. Neurological examination was normal. There is no history of PCD in the family (Fig. 1 d). The father has self-reported DD and one niece has been formally diagnosed with DD (Fig. 1 d). For an overview of the clinical phenotype, see Table 1.

**Table 1:** Clinical, behavioral and histological characterization of the individuals

Nr.	Nationality (Ethnic origin)	Gender	Age at time of study	Dyslexia	PCD	Laterality	Dynein arms	Neurological diagnoses	Heart	Vision	Spine	Handedness	Others
1	Sweden (Caucasian)	female	59	yes	yes	situs inversus totalis	lack of ODA, normal IDA	Asperger syndrome, ADHD, normal intellectual ability	normal	hypophoria, exophoria, otherwise normal	leftconvex thoracal scoliosis	left-handed	mirror writing ability
2	USA (Caucasian)	male	7	yes	biopsy inconclusive, not diagnosed	situs inversus totalis	inconclusive	hyperactive/ symptoms of ADHD, normal intellectual ability	normal	color-blind, otherwise normal	mild asymmetry of spine	right-handed	

*ODA* outer dynein arms*, IDA* inner dynein arms*, ADHD* attention deficit hyperactivity disorder

A DNA sample was isolated from saliva and was sequenced on one Illumina HiSeqX lane at an average sequencing depth of 32x (see Additional file 1, Methods). Due to the phenotypic triad of PCD, DD and scoliosis present in the individual, the analysis was first focused on *DYX1C1*, which has previously been associated to DD, causes PCD when mutated, and absence of the *dyx1c1* orthologue causes spine curves in zebrafish [15, 30, 31]. No SNVs that were previously associated to PCD or DD nor any other rare coding or noncoding variants were found in *DYX1C1*.

For downstream analysis, we extracted all the coding variants and canonical splice variants, excluded all synonymous variants and considered further only insertions, deletions, stop-gain, stop-loss and non-synonymous variants. The list was filtered to retain only variants with a minor allele frequency (MAF) of < 1% in the 1000G (all), 1000G (European), ExAC (total) and ExAC (non-Finnish European) databases. We focused on a set of PCD genes known to cause laterality defects (*n* = 33) [2, 4, 32] (Additional file 1, Table S1). As PCD is primarily inherited in autosomal recessive mode, we assumed homozygosity or compound heterozygosity. Therefore, biallelic variants were prioritized. We identified two nonsynonymous variants in *DNAH5,* which were confirmed by Sanger sequencing (Fig. 1 e, f). The variant c.7502G > C;p.(R2501P) (rs78853309; NC_000005.9:g.13810275C > G; NM_001369.2:c.7502G > C) in exon 45 has a frequency of 4 × 10^− 4^ in ExAC, 1.8 × 10^− 4^ in GnomAD and has not been reported in the 1000G and SweGen databases. It is predicted to be highly conserved (GERP = 5.31) or damaging in all assessed prediction tools (SIFT, Polyphen2, MutationTaster, CADD and GERP++) including a CADD score of 26.8. The same variant has previously been reported in two compound heterozygote patients with PCD [3, 33]. It is listed in ClinVar (ID:179699) and has been described as likely disruptive. It was classified as a Variant of Uncertain Significance (VUS) by the ACMG-AMP variant classification criteria [34]. The second variant, c.12043 T > G;p.(Y4015D) (rs754466516; NC_000005.9:g.13721345 T > G; NM_001369.2:c.12043 T > G) in exon 71, has a frequency of 1.65 × 10^− 5^ in ExAC, 0.81 × 10^− 5^ in GnomAD and has not been reported in the 1000G and SweGen databases. It is conserved (GERP = 5.4) and is predicted to be damaging by all the assessed prediction tools including a CADD score of 26.5. It has not previously been linked to PCD. Neither of the reported variants has been observed in a homozygous state in GnomAD. Parental DNA was not available for analysis; therefore these variants were analyzed in an unaffected sibling. The unaffected sibling is heterozygous for c.7502G > C and wildtype for c.12043 T > G (Fig. 1 d), suggesting that the affected individual is compound heterozygous for the *DNAH5* variants. To test whether *DNAH5* rare variants co-segregate with DD, targeted Sanger sequencing of the two nieces of the individual was performed, one affected and one unaffected with DD. None of them carry any of the two variants in *DNAH5* (Fig. 1 d). (For more in-depth sequence analysis see Additional file 1, Supplementary results.)

**Fig. 2:**
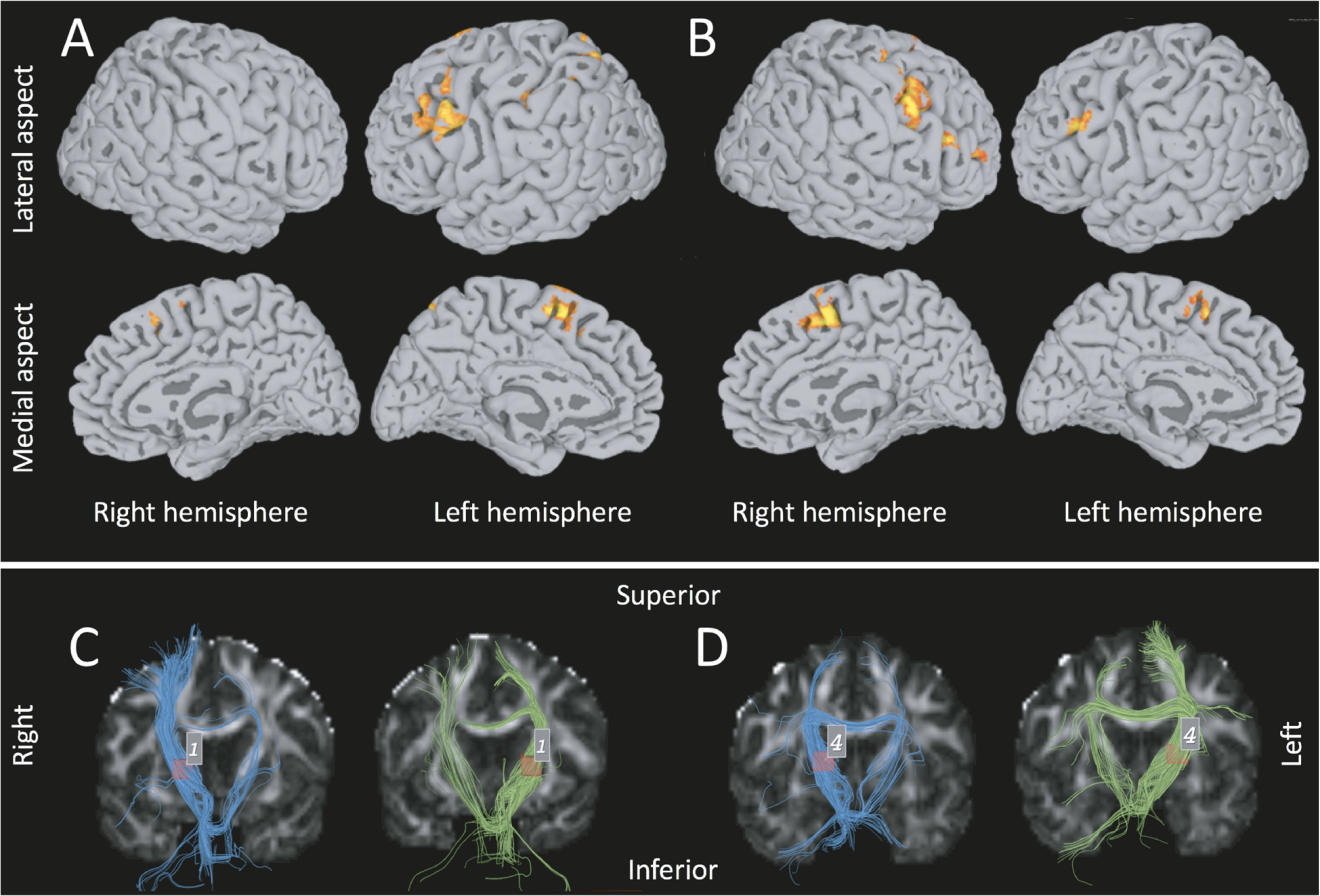
MRI Brain imaging individual 1. fMRI activations overlaid on the semi-inflated FreeSurfer cortical surface for individual 1 (**a**) and control (**b**) in response to the silent word generation task. DTI tractography of corticospinal tracts of individual 1 (**c**) and the matched control (**d**). Right hemisphere tracts in blue, left hemisphere tracts in green. Volume of interest in red

MRI brain scanning was performed to explore neuroanatomy and functionality in the presence of *situs inversus* and DD (see Additional file 1, Methods). Radiological assessment did not reveal any structural anatomical abnormalities in individual 1. The 3D T1-weighted images of individual 1 and a healthy control are presented in Additional file 1, Fig. S1. fMRI in combination with a silent word generation task was used to assess hemispheric laterality (Fig. 2 a, b). We observed a bilateral activation of frontal gyri in the control, which was absent in individual 1. Interestingly, there was additional activation of the parietal lobe in individual 1. Overall, these data do not allow conclusions about the hemispheric laterality of the individual. Diffusion tensor imaging (DTI) tractography of the corticospinal tracts was compared to an age- and gender-matchedright-handed control subject. In individual 1, we observed more DTI tracts on the right side than on the left side, which is reversed in relation to the control. In addition, we observed fewer corticospinal tracts crossing over the corpus callosum in individual 1 compared to the control (Fig. 2 c, d). Overall, the DTI data suggest a right-dominant hemisphere in the individual, possibly linked to the left-handedness.

In summary, we identified a previously known and a novel variant in *DNAH5*, as a likely cause for PCD in this individual.

### Case 2

**Fig. 3:**
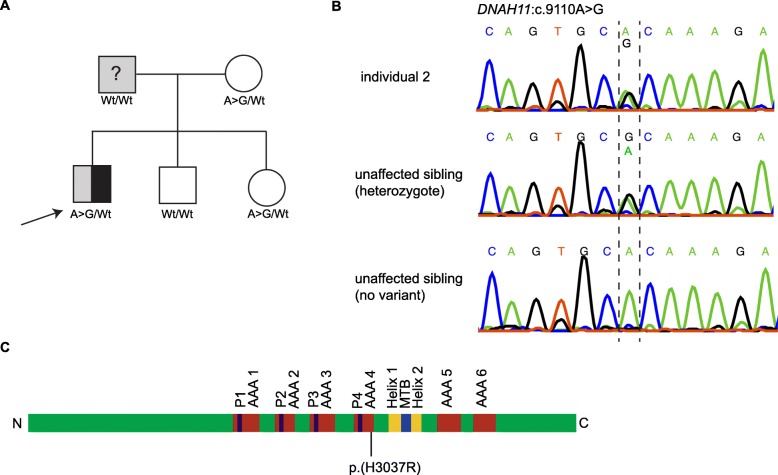
Genetics individual 2. **a** Pedigree. Individual 2 (arrow) has *situs inversus* (black) and DD (gray). The father has self-reported DD, but the dyslexia diagnosis is not confirmed (question mark). Unaffected individuals are shown in white. The genotypes of the variant in *DNAH11* are indicated (A > G denotes c.9110A > G, Wt denotes wildtype). **b** Sanger DNA sequencing chromatogram of individual 2 and controls (2 unaffected siblings). **c** Schematic representation of the domain structure of DNAH11 and localization of the amino acid substitution p.(H3037R). *N*=N-terminus, C=C-terminus, P1-P4 = P-loops 1–4, MTB = microtubule domain, AAA1–6 = AAA modules 1–6

Individual 2 is a Caucasian American boy affected by non-syndromic SI and DD (Gray Oral Reading Test 5 (GORT-5) overall reading index =84). He has a mild curvature of the lower thoracic spine towards the left, but no symptoms of PCD and a biopsy of the cilia was inconclusive (data not shown). His father has self-reported DD, but was not formally diagnosed (Fig. 3 a). The mother has mild scoliosis. There is no family history of SI or PCD. The individual also shows symptoms of ADHD. For an overview of the individual’s phenotypes, see Table 1.

DNA was extracted from a saliva sample and WGS was run on Illumina HiSeqX at a mean sequencing depth of 25x (see Additional file 1, Methods). First, we examined the *DYX1C1* gene and found the common haplotype -3G > A/1249G > T (rs3743205; rs57809907) previously associated to dyslexia [30]. However, the haplotype did not co-segregate with the dyslexia phenotype. It was also present in the mother and sibling, as revealed by Sanger sequencing (data not shown). We did not find any other SNVs that were previously associated to PCD or DD nor any other rare variants in *DYX1C1*.

As in individual 1, we extracted coding and canonical splice variants, excluded all synonymous variants and considered further only insertions, deletions, stop-gain, stop-loss and non-synonymous variants. Remaining variants were filtered by frequency MAF < 1% (see above) and compared to a list of genes associated with left-right defects (Additional file 1, Table S1). Individual 2 was heterozygous for a single rare variant c.9110A > G;p.(H3037R) (rs192327380; NC_000007.13:g.21813391A > G; NM_001277115.2:c.9110A > G) in *DNAH11* in exon 56, which we confirmed by Sanger sequencing (Fig. 3 b, c). The frequency of the variant is 1.5 × 10^− 3^ in ExAc, 0.8 × 10^− 3^ in 1000Genomes and 7.8 × 10^− 4^ in GnomAD. Targeted Sanger sequencing revealed that this variant was inherited from the mother (Fig. 3 a). The variant was predicted to be benign by all of the assessed prediction tools including a CADD score of 8.4. We then expanded our search to more common SNVs in *DNAH11* (MAF < 50%) but found none that were inherited from the father.

No disease-causing variants in genes known to cause L/R asymmetry defects (*n* = 39) [1] (Additional file 1, Table S1) were found. (For more in-depth sequence analysis see Additional file 1, Supplementary results.)

In summary, in individual 2 we identified a common DD-associated haplotype and a rare variant of unknown significance in the outer dynein arm component *DNAH11*, both inherited from the mother.

## Discussion and conclusions

Several reports have demonstrated that DD candidate genes have a role in cilia [8, 9, 12, 13, 15–19]. Furthermore, L/R asymmetry defects in the brain have been proposed as an anatomical basis to neurodevelopmental disorders such as schizophrenia and specifically to DD, possibly mediated by ciliary dysfunction [14, 28]. Here, we studied the underlying genetic causes in two individuals with DD and SI and/or PCD. This is, to our knowledge, the first study specifically addressing the genetics of DD co-occurring with a known ciliopathy.

In individual 1, we found one novel (c.7502G > C; p.(R2501P)) and one previously reported rare variant (c.12043 T > G;p.(Y4015D)) in *DNAH5.* Both variants are predicted to be damaging and c.7502G > C has been observed in at least two other PCD patients [3, 33]. We assume that individual 1 is probably compound heterozygous, as the likelihood of a recombination event or a de novo event is very low. The *DNAH5* gene encodes one of the outer arm axonemal dynein heavy chain proteins [3]. Mutations in *DNAH5* cause ODA defects, but do not affect IDAs, which is consistent with the ciliary ultrastructure found in the individual. Expression of *DNAH5* in the developing human brain is rather low as reported in the Allen Brain Atlas and it has no known function in primary cilia/ neuronal cilia (http://www.brainspan.org/) [35]. Possibly, *DNAH5* may lead to abnormal asymmetry in the brain via the left-right patterning via the cilia in the embryonic node. The asymmetry in the brain may then in turn contribute to DD. However, the genetic co-segregation pattern in the individual 1 pedigree shows that DD does not co-segregate with the variants in *DNAH5*. Alternatively, the DD inheritance pattern might be explained by an autosomal dominant inheritance pattern of a rare variant in another gene with incomplete penetrance in the sibling of the individual or by a complex inheritance pattern with several low penetrance common variants. WGS of all family members might clarify the inheritance pattern of DD, although the small size of the family complicates candidate-free approach WGS analysis.

The DTI tractography results suggest an inversion of hemispheric dominance, while fMRI language dominance testing was inconclusive. Abnormal symmetry of the brain - both increased asymmetry as well as decreased asymmetry – has been reported in dyslexics [6, 14, 36]. Whereas some studies report a typical left hemispheric language lateralization in the SI brain, others report a reversal of the language center to the opposite hemisphere [23–27]. While there is a robust association between handedness and hemispheric language dominance, the relationship between visceral situs and hemispheric dominance is more complex suggesting that brain asymmetry develops independently from the main symmetry-breaking pathway in the body [26, 37, 38]. The observed reversion of corticospinal fiber tracts may thus be likely related to the left-handedness of the individual.

In summary, we identified a previously known and a novel variant in *DNAH5*, as a likely cause for PCD in individual 1.

In individual 2, we identified a haplotype previously associated to DD. However, the haplotype did not co-segregate with the dyslexia phenotype. After careful examination of PCD and L/R-asymmetry genes, one rare variant in *DNAH11* was identified. A causative variant in the gene *DNAH11* is consistent with the normal ultrastructure of cilia in PCD patients with *DNAH11* mutations and the clinical report that the biopsy of cilia remained inconclusive. The lack of identification of the second variant does not exclude *DNAH11* as a candidate as there might be another yet unidentified genetic variant. However, the variant is predicted to be benign and *DNAH11* mutations have not been found in 13 cases of isolated *situs inversus* without PCD [39]. Furthermore, the variant c.9110A > G in *DNAH11* has been observed in a homozygous state in one individual in GnomAD. Overall, this evidence weakens the strength of *DNAH11* as a candidate in this individual. It should be noted that polygenic inheritance and also environmental factors have been suggested to play a role in laterality disorders which might explain the difficulty to find the causative variants in individual 2 [1]. In conclusion, we consider this case unsolved, in accordance with the observation that about 50% of SI cases without PCD remain unsolved after whole genome sequencing [40].

Taken together, we report a known and a novel variant in *DNAH5* as likely causative genetic variations for PCD that will be of value in the practice of diagnosing PCD. We believe that the reported variants are adding to the classification of VUSs. However, their involvement in DD pathology remains elusive. Regarding the DD phenotype, the possible role of these variants cannot be excluded but remains to be determined. Future functional studies should test specifically if the variants have functional consequences and aim at elucidating the role of ciliary genes on the brain in general. We propose the careful examination of variants in dynein/ciliary genes in individuals recruited for studies of DD.

## Supplementary information

**Additional file 1.** Supplementary Results; Methods; Table S1: PCD and L/R genes used for filtering; Fig. S1: Structural MRI findings.

## Data Availability

The datasets generated and analyzed during the current study are withheld for confidentiality reasons, but can be made available to qualified researchers by reasonable request to the corresponding authors.

## Abbreviations

ADHD: Attention deficit hyperactivity disorder
DCDC2: Doublecortin domain containing 2
DD: Developmental dyslexia
DNAH11: Dynein axonemal heavy chain 11
DNAH5: Dynein axonemal heavy chain 5
DTI: Diffusion tensor imaging
DYX1C1/DNAAF4: Dyslexia susceptibility 1 candidate 1; Dynein axonemal assembly factor 4
fMRI: Functional magnetic resonance imaging
L/R: Left-right
MRI: Magnetic resonance imaging
PCD: Primary ciliary dyskinesia
SI: Situs inversus
SNV: Single nucleotide variation
VUS: Variant of uncertain significance
WGS: Whole genome sequencing
