# Supplementary material for: Rare variants in dynein heavy chain genes in two individuals with situs inversus and developmental dyslexia: a case report
Source: BMC Med Genet. 2020 May 1;21:87. doi: 10.1186/s12881-020-01020-2 (PMC7193346; doi:10.1186/s12881-020-01020-2)
Supplement: Supplementary file 1 — Additional file 1. Supplementary Results; Methods; Table S1: PCD and L/R genes used for filtering; Fig. S1: Structural MRI findings. [file 12881_2020_1020_MOESM1_ESM.docx]

**Additional file**

***Supplementary Results***

**Case 1**

Upon examination of the known dyslexia susceptibility variants, we only found variants that are common in the general population. In addition, an unbiased approach filtering by CADD score and retaining all variants with a score >25 did not reveal any additional plausible variants. CNVs have been reported in PCD patients and in heterotaxia (1, 2) and as potential risk factors for DD (3, 4). Structural variant analysis of the WGS data revealed one inversion encompassing *CCDC103*, a gene implicated in PCD. We did not find any other structural variants in regions overlapping with candidate genes.

**Case 2**

When examining the known dyslexia susceptibility variants we only found variants that are common in the general population. An unbiased approach filtering all the variants with a CADD score >25 did not reveal any additional interesting variants supporting the phenotypes of individual 2. Structural variant analysis revealed one CNV within *NEGR1*, a gene in which a CNV previously has been associated with DD (3). We did not find any other structural variants in regions overlapping with candidate genes.

We further checked a list of intronic variants close to exons (≤20bp), but did not find any rare variants. In addition, we looked for predicted splice effects of rare *DNAH11* deep intronic variants. We found a possible creation of a new acceptor site at chr7:21809845 (NC_000007.13:g.21809845T>G; NM_001277115.1:c.9103-3539T>G; heterozygous variant), however this variant is common (GnomAD= 6.1x10^-2^) and probably not contributing to the phenotype.

There has been a report on digenic inheritance of PCD with *situs solitus* via *DNAH11* and *DNAH2* (5). One rare, nonsynonymous, missense variant in *DNAH2* c.2495A>G:p.(Q832R) (rs181090270; NC_000017.10:g.7646805A>G; NM_001303270:c.2495A>G) was identified. It has a frequency of 1.6x10^-3^ in the 1000Genome database, 1.5x10^-3^ in GnomAD and is non-existent in ExAC; it is predicted to be benign with a CADD score of 7.167.

***Methods***

**Electron microscopy**

Epithelial cells from the nasal cavity were collected and prepared using standard procedures. Digital images were captured with a FEI Tecnai BioTwin transmission electron microscope (FEI Inc., Eindhoven, Netherlands) equipped with a Gatan Orius SC1000 CCD camera (Gatan Inc., Abingdon, United Kingdom). Data are expressed as mean +/- SEM.

**Whole-genome sequencing and sequence analysis**

Saliva samples were collected using Oragene DNA kits (OG-500; DNA Genotek, Ottawa, Canada) and genomic DNA from saliva was extracted using the prepIT PT-L2P (DNA Genotek) according to the manufacturer´s instructions. DNA samples were sequenced and analyzed using standard procedures (human genome build GRCh37). The databases used for filtering were the 1000 Genomes project (accessed June/September 2017) and the Exome aggregation consortium (accessed June/September 2017). The GnomAD database, and for individual 1, additionally, the SweGen database (6), were consulted for frequency (accessed July 2018). The impact of variants was evaluated using the prediction tools SIFT, Polyphen2, MutationTaster, CADD and GERP++. For splice effect prediction, SeattleSeqAnnotation138 annotation (7) and AlamutVisual 2.11 splicing prediction module (8) were used. Copy number variants (CNVs) were called using the CNVnator v.0.3.2 (9) and both CNVs and balanced structural variants (SV) were identified using TIDDIT 2.2.3 (10). Variants were filtered by frequency using the SweGen database and included all SVs at a frequency of ≤0.001. The remaining SNVs and SVs were compared to candidate gene lists (Additional file Table S1). Sanger sequencing of PCR products was used to validate selected WGS variants.

**Magnetic resonance imaging (MRI)**

Individual 1 and a healthy age-matched female control (59 years old, normal-reader, right-handed) were studied. All MRI scans were conducted on a whole-body 3T Prisma^Fit^ clinical MRI scanner (Siemens, Erlangen, Germany) using a 64-channel head coil. Both the fMRI and DTI scanning protocols were adapted to the acquisition protocols in the Human Connectome Project with 2 mm isotropic voxel size ([www.humanconnectomeproject.org](http://www.humanconnectomeproject.org)). Standard clinical imaging sequences were acquired. The task-based fMRI consisted of three runs of word generation conducted using a block design. The DTI session included 3 runs (each lasting approximately 7 minutes), representing 3 different gradient tables. The gradient tables were identical to those used for the Human Connectome Project.

**References**

1. Marshall CR, Scherer SW, Zariwala MA, et al. Whole-Exome Sequencing and Targeted Copy Number Analysis in Primary Ciliary Dyskinesia. G3 (Bethesda). 2015;5(8):1775-81.

2. Cao R, Long F, Wang L, et al. Duplication and deletion of CFC1 associated with heterotaxy syndrome. DNA Cell Biol. 2015;34(2):101-6.

3. Veerappa AM, Saldanha M, Padakannaya P, Ramachandra NB. Family based genome-wide copy number scan identifies complex rearrangements at 17q21.31 in dyslexics. Am J Med Genet B Neuropsychiatr Genet. 2014;165b(7):572-80.

4. Gialluisi A, Visconti A, Willcutt EG, et al. Investigating the effects of copy number variants on reading and language performance. J Neurodev Disord. 2016;8:17.

5. Berg JS, Evans JP, Leigh MW, et al. Next generation massively parallel sequencing of targeted exomes to identify genetic mutations in primary ciliary dyskinesia: implications for application to clinical testing. Genet Med. 2011;13(3):218-29.

6. Ameur A, Dahlberg J, Olason P, et al. SweGen: a whole-genome data resource of genetic variability in a cross-section of the Swedish population. Eur J Hum Genet. 2017;25(11):1253-60.

7. Ng SB, Turner EH, Robertson PD, et al. Targeted capture and massively parallel sequencing of 12 human exomes. Nature. 2009;461(7261):272-6.

8. Houdayer C. In silico prediction of splice-affecting nucleotide variants. Methods Mol Biol. 2011;760:269-81.

9. Abyzov A, Urban AE, Snyder M, Gerstein M. CNVnator: an approach to discover, genotype, and characterize typical and atypical CNVs from family and population genome sequencing. Genome Res. 2011;21(6):974-84.

10. Eisfeldt J, Vezzi F, Olason P, Nilsson D, Lindstrand A. TIDDIT, an efficient and comprehensive structural variant caller for massive parallel sequencing data. F1000Res. 2017;6:664.

**Table S1: PCD and L/R asymmetry genes**

| **PCD genes causing laterality defects** | |  | **L-R asymmetry genes (excluding PCD genes)** | |
| --- | --- | --- | --- | --- |
| *AK7* | Mata et al., 2012 |  | *ACVR2B* | Deng et al., 2014 |
| *ARMC4* | Knowles et al., 2016 |  | *ANKS6* | Deng et al., 2014 |
| *C21ORF59 (CFAP298)* | Knowles et al., 2016 |  | *ARL2BP* | Deng et al., 2014 |
| *CCDC103* | Knowles et al., 2016 |  | *BBS2* | Deng et al., 2014 |
| *CCDC11* | Narasimhan et al., 2015 |  | *BBS8* | Deng et al., 2014 |
| *CCDC114* | Knowles et al., 2016 |  | *C1ORF88* | Deng et al., 2014 |
| *CCDC151* | Knowles et al., 2016 |  | *CEP290* | Deng et al., 2014 |
| *CCDC39* | Knowles et al., 2016 |  | *CFC1* | Deng et al., 2014 |
| *CCDC40* | Knowles et al., 2016 |  | *CITED2* | Deng et al., 2014 |
| *CFAP300 (C11ORF70)* | Fassad et al., 2018a, Höben et al., 2018 |  | *CRELD1* | Deng et al., 2014 |
| *DNAAF1* | Knowles et al., 2016 |  | *EPB41L5* | Deng et al., 2014 |
| *DNAAF2* | Knowles et al., 2016 |  | *FOXH1* | Deng et al., 2014 |
| *DNAAF3* | Knowles et al., 2016 |  | *GALNT11* | Deng et al., 2014 |
| *DNAAF5 (HEATR2)* | Knowles et al., 2016 |  | *GATA4* | Deng et al., 2014 |
| *DNAH1* | Knowles et al., 2016 |  | *GDF1* | Deng et al., 2014 |
| *DNAH11* | Knowles et al., 2016 |  | *HES7* | Deng et al., 2014 |
| *DNAH5* | Knowles et al., 2016 |  | *INVS* | Deng et al., 2014 |
| *DNAH6* | Li et al., 2016 |  | *LEFTY2* | Deng et al., 2014 |
| *DNAH8* | Knowles et al., 2016 |  | *LZTFL1* | Deng et al., 2014 |
| *DNAH9* | Fassad et al., 2018b, Loges et al., 2018 |  | *MED13L* | Deng et al., 2014 |
| *DNAI1* | Knowles et al., 2016 |  | *MEGF8* | Deng et al., 2014 |
| *DNAI2* | Knowles et al., 2016 |  | *MMP21* | Perles et al., 2015 |
| *DNAL1* | Knowles et al., 2016 |  | *NEK2* | Deng et al., 2014 |
| *DYX1C1 (DNAAF4)* | Knowles et al., 2016 |  | *NEK8* | Deng et al., 2014 |
| *FOXJ1* | Wallmeier et al., 2019 |  | *NKX2.5* | Deng et al., 2014 |
| *LRRC56* | Bonnefoy et al., 2018 |  | *NODAL* | Deng et al., 2014 |
| *LRRC6* | Knowles et al., 2016 |  | *NPHP3* | Deng et al., 2014 |
| *NME8 (TXNDC3)* | Knowles et al., 2016 |  | *NPHP4* | Deng et al., 2014 |
| *OFD1* | Knowles et al., 2016 |  | *NUP188* | Deng et al., 2014 |
| *PIH1D3* | Olcese et al., 2017 |  | *PKD1L1* | Vetrini et al., 2016 |
| *SPAG1* | Knowles et al., 2016 |  | *PKD2* | Deng et al., 2014 |
| *TTC25* | Wallmeier et al., 2016 |  | *ROCK2* | Deng et al., 2014 |
| *ZMYND10* | Knowles et al., 2016 |  | *SESN1* | Deng et al., 2014 |
|  |  |  | *SHROOM3* | Deng et al., 2014 |
|  |  |  | *SMAD2* | Deng et al., 2014 |
|  |  |  | *TGFBR2* | Deng et al., 2014 |
|  |  |  | *UVRAG* | Deng et al., 2014 |
|  |  |  | *WDR16* | Ta-Shma et al., 2015 |
|  |  |  | *ZIC3* | Deng et al., 2014 |

***
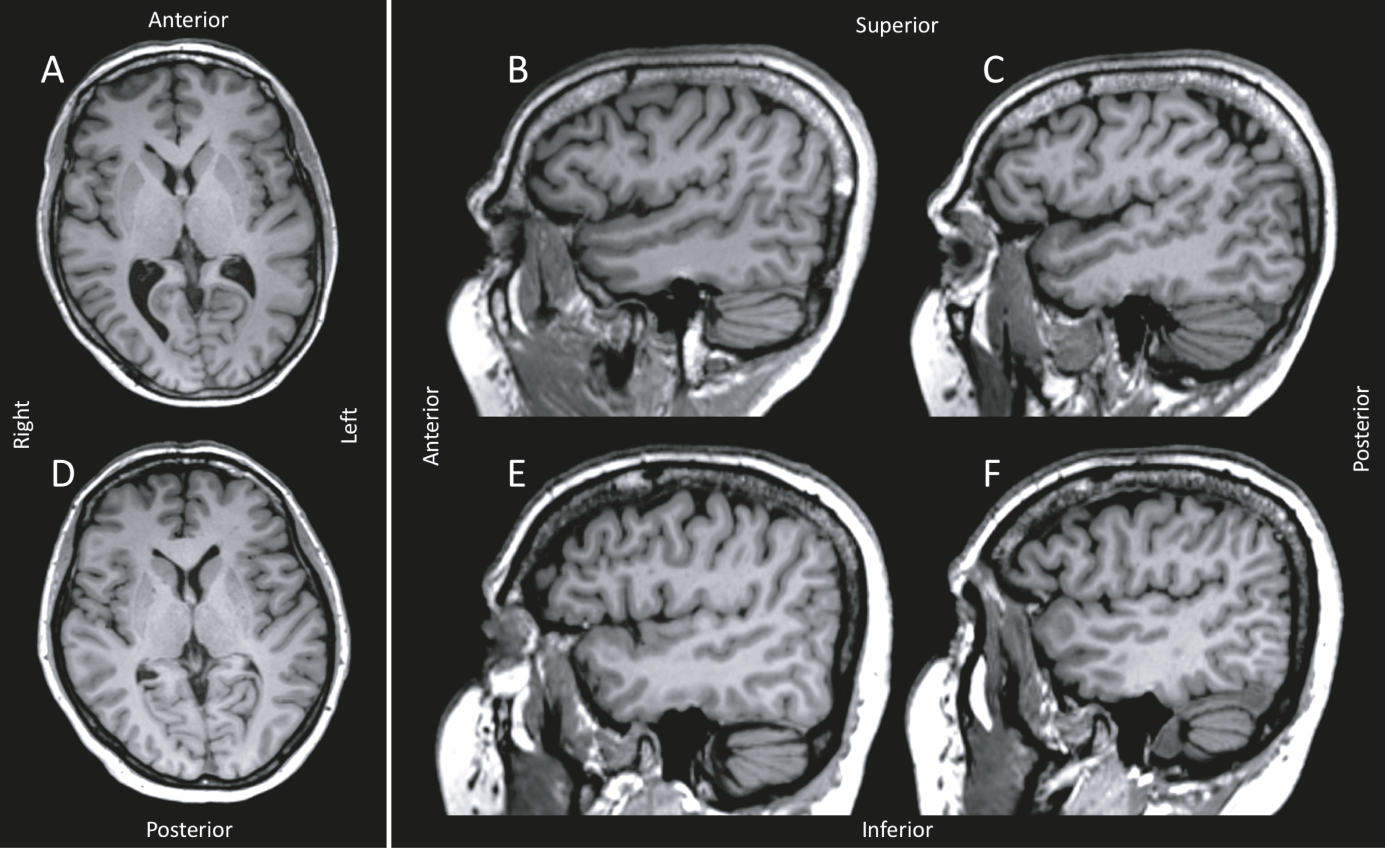
***

**Fig. S1: Structural MRI findings.** Axial (A,D) and sagittal (B,C,E,F) 3D T1-weighted images of individual 1 (A-C) and the matched healthy control (D-F). Radiological readings did not reveal any significant structural anatomical anomalies.
